# Unified and distinct cognitive control deficits in adolescents with cognitive disengagement syndrome and learning burnout

**DOI:** 10.3389/fpsyt.2024.1399122

**Published:** 2024-07-03

**Authors:** Yuhan Weng, Tingting Wu, Yunfang Wei, Ling Chen, Xiqin Liu, Kexin Cai, Caiqi Chen

**Affiliations:** ^1^ School of Psychology, South China Normal University, Guangzhou, China; ^2^ Key Laboratory of Brain, Cognition and Education Sciences, Ministry of Education, South China Normal University, Guangzhou, China; ^3^ Center for Studies of Psychological Application, South China Normal University, Guangzhou, China; ^4^ Guangdong Key Laboratory of Mental Health and Cognitive Science, South China Normal University, Guangzhou, China; ^5^ Beijing Key Lab of Learning and Cognition, School of Psychology, Capital Normal University, Beijing, China; ^6^ Shantou Special Economic Zone Linbaixin Middle School, Swatow, China; ^7^ School of Foreign Languages, South China University of Technology, Guangzhou, China

**Keywords:** cognitive disengagement syndrome, sluggish cognitive tempo, learning burnout, attentional network, cognitive control, adolescents

## Abstract

**Introduction:**

Cognitive disengagement syndrome (CDS) is a psychological disorder characterized by daydreaming, mental fogginess, and slow thinking, while learning burnout (LB) is characterized by a passive and inattentive attitude toward learning. These two disorders are closely related but can be challenging to differentiate from one another. The present study aimed to identify shared and distinct cognitive control deficits between CDS and LB.

**Methods:**

We recruited 136 adolescents (aged 14 to 17 years) from an initial screening of CDS and LB (N = 476) and divided them into four groups: CDS, LB, CDS + LB, and typically developing control. After a second screening, 129 adolescents completed two tasks to assess their attentional networks and cognitive control capacity (CCC).

**Results:**

Adolescents with high CDS symptoms (both CDS group and CDS+LB group) exhibited impaired disengaging effect of attention and lower CCC, indicating deficits in orienting attention and the upper limit of information processing for cognitive control specifically. Furthermore, support vector machine modeling identified CCC as the most significant parameter differentiating the CDS and LB groups.

**Discussion:**

Our findings suggest that while adolescents with high CDS and high LB symptoms have similar outward manifestations in the adolescent’s school life, deficits in attention and cognitive control, particularly in the CCC, may distinguish between the two groups.

## 1 Introduction

Cognitive disengagement syndrome (CDS), formerly known as sluggish cognitive tempo (SCT), is a psychological disorder characterized by persistent cognitive deficits across contexts, such as daydreaming, slow mental and physical activity, as well as difficulties in emotional regulation, social functioning, and academic performance (1, 2). The renaming from SCT to CDS aimed to highlight the core cognitive deficits and move away from the more metaphorical description implied by “sluggishness.” As a trait-like disorder (3), CDS is a pervasive condition that impacts various aspects of an individual’s daily life beyond academic settings. Moreover, evidence suggests that psychiatrists suffering from CDS may have a higher propensity for general clinical burnout symptoms later in life (4). In academic settings, however, symptoms of CDS may sometimes be misattributed to learning burnout (LB) in adolescents (5). LB, on the other hand, refers to a more temporary, context-specific status characterized by passive and inattentive attitudes specifically toward learning and learning-related activities in school settings (6). Unlike the metaphorical use of “burnout” to describe general fatigue or tiredness, LB is a distinct construct that captures the diminished drive, disengagement, and inattentiveness exhibited by students toward their academic pursuits. Students with LB exhibit decreased interest and motivation in learning, leading to boredom, fatigue, and depression symptoms (7). However, their functioning remains relatively normal in non-academic domains. Although CDS and LB have some similarities such as inactivity during learning and academic impairment (8–10), they differ fundamentally in their essential characteristics. CDS represents an enduring, cross-situational cognitive deficit, whereas LB is a more transient state confined to academic contexts (6). This key difference results in varying levels of symptom severity, chronicity, and degree of impairment in daily functioning between the two conditions. Additionally, CDS often requires pharmacological interventions (11, 12), while LB typically responds better to non-pharmacological approaches, such as mindfulness education and progressive muscle relaxation (13). Clearly differentiating the theoretical underpinnings of trait-like CDS and state-like LB not only aids in better interpreting empirical findings through the lens of their respective theoretical frameworks but also informs intervention strategies for these cognitive deficit conditions from a theoretical perspective. However, due to symptom similarity, distinguishing adolescents with CDS from LB can be difficult.

Cognitive control may be critical to differentiate between CDS and LB. Extensive research on CDS has identified varying degrees of impairment in cognitive control functions (1, 14–18), which are general executive abilities essential for goal-directed behaviors and information processing under conditions of uncertainty (19–21). In contrast, the relationship between non-clinical burnout symptoms and cognitive control deficits remains unclear (4, 22–28). Cognitive control involves interactive attention networks such as alerting, orienting, executive control, supporting the selection and prioritization of goal-relevant information. It also serves as a core construct of broader executive functions, including inhibitory control, working memory updating, and cognitive flexibility (21, 29). Findings suggest CDS individuals may have difficulty selecting and directing attention resource to goal-relevant information, exhibiting as deficits in alerting and orienting networks (14–17). CDS may also have deficits in the ability to carry out goal-directed behavior and resolve conflicts purposefully, exhibiting as impaired efficiency in executive control of the network (16). However, non-significant associations between CDS and attention networks have also been reported in juvenile (30, 31). Moreover, CDS populations also exhibit deficits in broader executive functions, such as poor inhibitory control (32), working memory (33), and cognitive flexibility (14), which may partly stem from alerting or/and orienting attention problems (34, 35).

Another key catachrestic of cognitive control is its severely limited capacity. Due to its role as a core construct bridging the selection of information and further process by higher-order cognitive functions, cognitive control capacity (CCC) not only restricts the information processing efficiency of cognitive control per se, but also generally limits the processing efficiency of broader functions that involves cognitive control, and even intellectual ability (36, 37). The core “sluggishness” deficit in CDS is potentially attributable to impaired CCC, which can also explain its broad, persistent cognitive deficits across contexts. Our previous studies have developed a behavioral assessment quantifying CCC as the maximum amount of information processing that can be accurately processed by cognitive control per second (37), which can be used to directly test this proposal. Examining CDS and LB in relation to CCC could elucidate key distinctions in their impact on domain-general vs. context-specific cognitive functions.

For burnout, most studies have focused on occupation burnout rather than learning burnout (4). While some findings link burnout to diminished attentional ability (22) and poor executive functioning (22–25), others report no significant associations, especially for milder, non0clinical burnout symptoms (26–28). This discrepancy suggests that unlike CDS, learning burnout (LB) may not necessarily involve general cognitive control deficits but could represent a more context-specific response (6).

The present study aims to investigate the deficits of CDS and LB in adolescents with a focus on cognitive control. Four participants groups were included: (1) adolescents with high CDS but not high LB (CDS group), (2) adolescents with high LB but not high CDS (LB group), (3) adolescents with composite symptoms of CDS and LB (CDS + LB group), and (4) typically developing control (TD) group. Cognitive control abilities were evaluated by measuring the efficiency of attentional networks and cognitive control capacity (CCC). We hypothesized that adolescents with high CDS symptoms would exhibit impairments in cognitive control, specifically with deficits in the executive control functions of attention and lower CCC, while adolescents with high LB symptoms would not show those impairments. This investigation may help to clarify the cognitive deficits that are unique or shared between CDS and LB individuals.

## 2 Methods

### 2.1 Participants

A total of 476 adolescents (aged 14 to 17 years, 286 females and 190 males) from high schools in Swatow, China, participated in the initial screening. They completed two questionnaires in Chinese: Child Concentration Inventory - Version 2 (CCI-2) (38) and Adolescent Student Burnout Inventory (ASBI) (39). High CDS level was defined as scoring five or more items rated 3 on the CCI-2 based on literature (5, 17), as these participants exhibited at least five CDS symptoms over the past six months. High LB level was defined as an ASBI score higher than 70% of the total score (≥ 56). This standard for LB was based on commonly used criteria for statistical analysis due to the lack of cognitive experiments conducted on the LB group. Given that our study participants were drawn from a key middle-high school where student achievements are relatively high, it is reasonable to conclude that LB levels were relatively low. In this context, viewing the upper 30% of the initial screening as high LB may result in bias. In light of the sample characteristics and the questionnaire-based criteria for CDS, the upper 30% of the total ASBI score was considered to represent the high LB level. Following these criteria, 136 adolescents proceeded to the follow-up experiments, categorized into four groups: (1) CDS group (high in CDS only), (2) LB group (high in LB only), (3) CDS + LB group (high in both CDS and LB), and (4) TD group (neither high in CDS nor LB).

Seven participants were excluded from the analysis of Attention Network Task-Revised (ANT-R) due to inadequate attention, evidenced by non-responsiveness or incorrect responses in over 50% of the trials. Additionally, two participants were excluded from the analysis of the Backward-Masking Majority Function Task (MFT-M) due to insufficient task engagement, reflected by an accuracy below 70% in the easiest condition. Moreover, seventeen participants opted out of the MFT-M task due to personal reasons. Consequently, the final sample consisted of 129 participants who completed the ANT-R and 117 who completed the MFT-M. Of these, 86 participants who completed both tasks were included in the subsequent analysis using the support vector machine (SVM) models. Demographic details and CCI-2/ASBI scores for each group were reported in **Table 1**.

**Table 1:** Demographic information, and mean ± standard deviation of the CCI-2 and ASBI scores for adolescents participated in each task.

	CDS	LB	CDS + LB	TD	*F*/*χ^2^*	*p*	*BF_10_*
ANT-R							
N	29	34	29	37			
Male: Female (Sex ratio)	11:18 (1:1.64)	8:26 (1:3.25)	11:18 (1:1.64)	14:23 (1:1.64)	2.3	.513	0.105
Age (year-old)	15.4 ± 0.6	15.2 ± 0.5	15.2 ± 0.4	15.4 ± 0.5	2.16	.096	0.485
CCI-2	34.2 ± 5.4	23.0 ± 4.6	36.8 ± 5.4	14.5 ± 5.0	136.28	<.001^***^	> 1000
ASBI	47.7 ± 5.5	60.0 ± 4.2	63.2 ± 6.5	34.8 ± 9.5	122.34	<.001^***^	> 1000
MFT-M							
N	30	23	26	38			
Male: Female (Sex ratio)	11:19 (1:1.73)	7:16 (1:2.29)	10:16 (1:1.60)	14:24 (1:1.71)	< 1	.941	0.043
Age (year-old)	15.4 ± 0.5	15.2 ± 0.5	15.3 ± 0.5	15.4 ± 0.6	1.6	.195	0.293
CCI-2	35.0 ± 5.7	23.8 ± 3.7	36.4 ± 5.7	13.4 ± 5.2	139.00	<.001^***^	> 1000
ASBI	47.6 ± 5.1	60.5 ± 6.9	63.9 ± 6.9	35.9 ± 8.0	122.66	<.001^***^	> 1000
SVM							
N	24	20	22	20			
Male: Female (Sex ratio)	8:16 (1:2.00)	10:12 (1:1.20)	7:13 (1:1.86)	6:14 (1:2.33)	1.25	.742	0.1
Age (year-old)	15.4 ± 0.5	15.2 ± 0.5	15.2 ± 0.4	15.6 ± 0.6	1.5	.221	0.307
CCI-2	34.9 ± 5.0	23.6 ± 3.8	36.7 ± 5.8	14.5 ± 5.2	89.70	<.001^***^	> 1000
ASBI	47.5 ± 4.8	60.8 ± 4.3	64.3 ± 7.0	39.8 ± 7.7	73.53	<.001^***^	> 1000

N, number of participants; CCI-2, Child Concentration Inventory - Version 2; ASBI, Adolescent Student Burnout Inventory; CDS, cognitive disengagement syndrome; LB, learning burnout; TD, typically developing peers; ANT-R, participants who completed the Attention Network Task-Revised; MFT-M, participants who completed the backward-masking majority function task; SVM, participants involved in the support vector machine analysis. ***: p <.001.

### 2.2 Scales

The CDS level was assessed by employing the CCI-2, a self-report scale containing 15 items used for children aged 8-17 years (38). Participants rated their experiences in the past six months using a four-point scale ranging from 0 (never) to 3 (always). Prior to use in this study, the CCI-2 was translated into Chinese by three psychology researchers and revised by two English professors. The translation was then reviewed by two psychologists to ensure the accuracy of the Chinese version. The internal consistency of the Chinese version of the CCI-2 was high, with a Cronbach’s α coefficient of 0.92.

The LB level was assessed by employing the ASBI, a scale consisting of 16 items, which can be divided into three dimensions: Exhaustion, Learning cynicism, and Reduced efficacy (39). Participants were asked to rate their experiences over the past six months on a five-point scale ranging from 1 (completely inconsistent) to 5 (completely consistent). Higher scores on the ASBI indicate higher levels of LB. The Cronbach’s α coefficient of the entire scale was 0.85 in the present study, indicating good internal consistency.

Both CDS and LB were assessed through self-report questionnaires, as self-reports have been found to better predict long-term outcomes and cognitive correlates of CDS compared to parent/teacher reports (40, 41). The self-report approach also ensured consistency between the CDS and LB measures, with the most widely used Chinese questionnaire for LB also being a self-report measure. To reduce potential underreporting, participants were assured that their responses would not impact their academic standing.

### 2.3 Tasks

**Figure 1:**
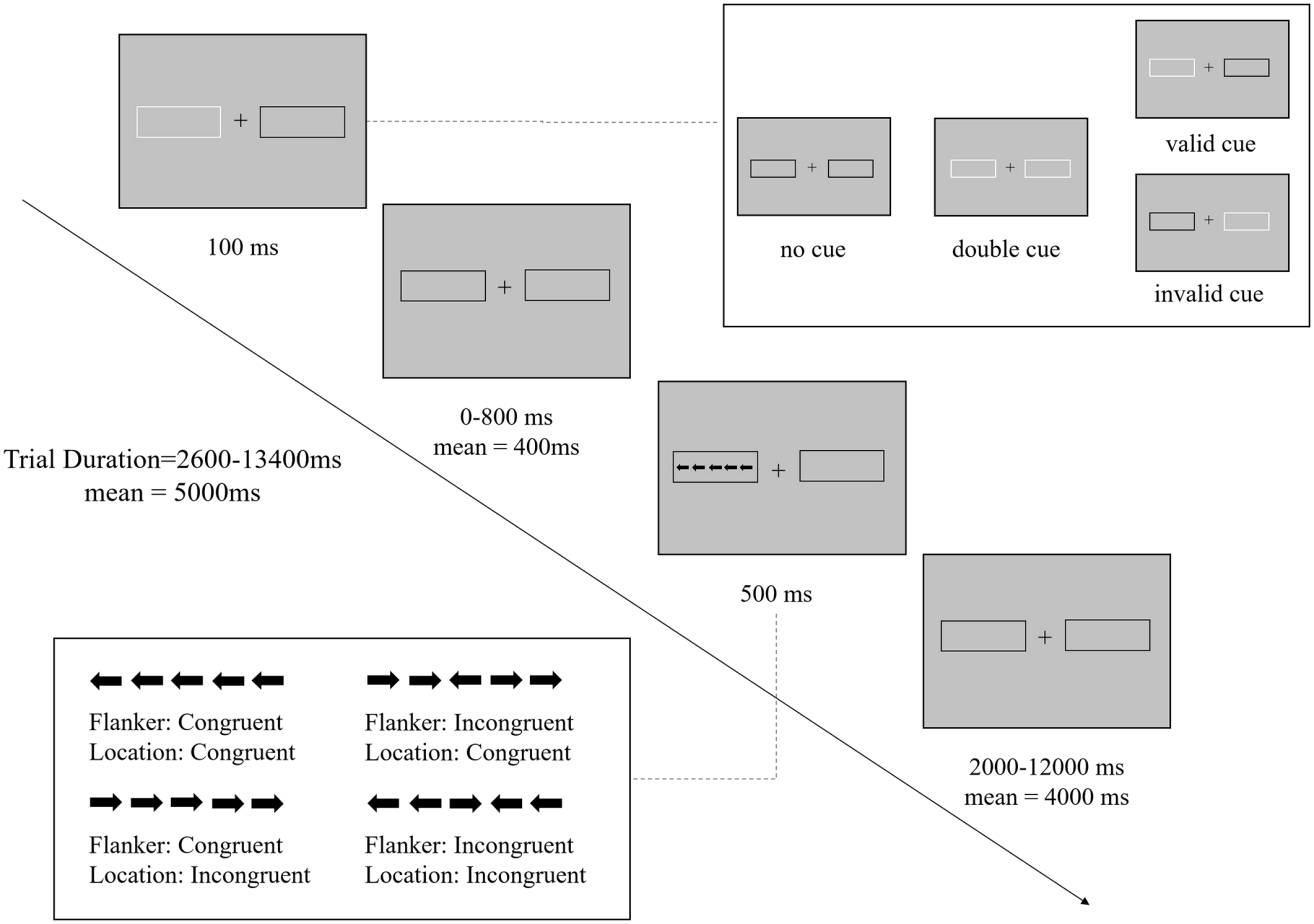
Procedure of the Attentional Network Task-Revised. Schematic overview of the trial structure and timings (example of a trial in congruent condition). There are four cue conditions: no cue, double cue, valid cue, invalid cue; and four conflict conditions: Flanker congruent and Location congruent, Flanker congruent and Location incongruent, Flanker incongruent and Location congruent, Flanker incongruent and Location incongruent.

The efficiency of the three attention networks—alerting, orienting, and executive control—was assessed using the Attention Network Task-Revised (ANT-R) adapted from the original ANT by Fan et al. (42, 43). Each trial of the ANT-R followed a timeline depicted schematically in **Figure 1**. Initially, participants were presented with a fixation presenting at the screen’s center with a box on each side for 100 ms. During the task, participants experienced different cueing conditions. In the no cue condition, no cue was presented before the task stimulus, serving as the baseline condition. Alternatively, in the double cue condition, both boxes flashed simultaneously, providing only temporal information about the impending task stimulus. The spatial cue condition involved a cue flashing in one of the boxes, providing both temporal and spatial information about the target location. This cue could be either valid (matching the subsequent target location) or invalid (mismatching the subsequent target location). Following a 0 to 800 ms (mean 400 ms) interval, a row of arrows was presented in one of the boxes as the task stimulus for 500 ms. Each trial ended with a 2000 to 12000 ms (mean 4000 ms) interval and lasted 2600 to 13400 ms (mean 5000 ms) in total. Participants were instructed to respond as quickly and accurately as possible to the target arrow’s direction (i.e., the arrow in the middle of the stimuli set) using designated keys (“F” for left and “J” for right). The ANT-R employed a 4 (Cue: no cue, double cue, valid cue, invalid cue) × 2 (Flanker conflict: congruent, incongruent) × 2 (Location conflict: congruent, incongruent) factorial design, yielding in 16 conditions. As illustrated in **Figure 1**, Flanker congruency referred to whether the flanker arrows pointed in the same direction as the target (congruent) or to the opposite direction (incongruent); Location congruency indicated whether the target pointed to the same direction as the location of the task stimulus (congruent) or to the opposite direction (incongruent). A total of 144 trials were presented randomly across two blocks, with each block containing all conditions, and the experiment lasted approximately 30 minutes.

**Figure 2:**
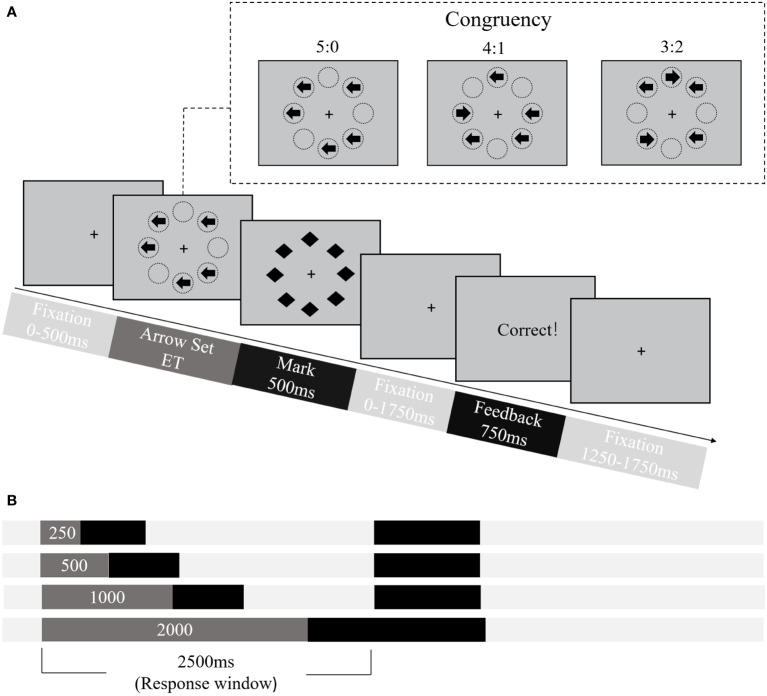
Procedure of the Backward-Masking Majority Function Task (MFT-M). **(A)** Schematic overview of the trial structure and timings. Stimuli set consisted of five arrows and formed three levels of congruency, i.e., the ratio between number of trials pointed to the majority versus minority direction: 5:0, 4:1, 3:2. *ET*, exposure time. **(B)** Illustration of four levels of exposure time (areas in dark gray).

To estimate CCC in terms of the maximum amount of information that can be accurately processed by cognitive control per unit of time, the backward-masking majority function task (MFT-M) was utilized (37). Each MFT-M trial timeline was schematic illustrated in **Figure 2A**. Initially, a fixation point appeared for 0-500 ms, followed by a set of five left/right-pointing arrows that appeared randomly at five of the eight octagonal locations surrounding the central fixation for a varied exposure time (ET). After the ET, a backward masking of eight black diamond shapes covering all eight locations was displayed for 500 ms, followed by a varied fixation period. Participants were asked to identify the majority direction of the arrows by pressing either “F” for left or “J” for right. While participants were encouraged to respond on every trial within a 2500 ms window, even if they were unsure about the correct answer, they were also instructed to prioritize accuracy over speed. The ratio of majority to minority direction arrows varied across three levels (5:0, 4:1, 3:2), while the ET ranged across four levels (250, 500, 1000, or 2000 ms) (**Figure 2B**). Feedback on response accuracy was presented at the end of the trial. Each trial lasted 5750 ms. The MFT-M employed a 3 (Ratio: 5:0, 4:1, 3:2) × 4 (ET: 250 ms, 500 ms, 1000 ms, 2000 ms) factorial design, resulting in 12 conditions. The task consisted of 8 blocks in the test, each with 36 trials of a single ET randomized across blocks, while the ratio varied with each block. The entire task was approximately 30 minutes.

### 2.4 Statistical analysis

Reaction time (RT) and accuracy were computed and analyzed using MATLAB 2019b (RRID: SCR_001622) and SPSS 22.0 (RRID: SCR_002865). Trials with no response were deemed incorrect and excluded from RT analysis. Accuracy in each condition was determined by calculating the proportion of trials of correct response. Additionally, trials with an RT exceeding three standard deviations (SD) of the mean across correct response trials in each condition were also excluded from RT analysis. The mean RT of each condition was then calculated based on the remaining trials within that corresponding condition. RT and accuracy were averaged across all conditions as overall performance.

The attention network effects for the ANT-R were computed by subtracting conditions (42): Alerting effect = no cue minus double cue; Orienting effect = double cue minus valid cue; Validity effect = invalid cue minus valid cue; Disengaging effect = invalid cue minus double cue; Flanker conflict = flanker incongruent minus flanker congruent; Location conflict = location incongruent minus location congruent. Greater attention network effect in RT indicates lower processing efficiency of the corresponding network. Attention network effects were computed for both RT and accuracy.

The CCC was estimated by fitting the model of the relationship between the increase of information rate and response accuracy declines in the MFT-M, as proposed by Wu et al. (37). The CCC, measured in bits per second (bps), represents the maximum amount of information that can be accurately processed by cognitive control in a unit of time.

A 4-level (Group: CDS, LB, CDS + LB, TD) one-way ANOVA tested differences in each attention network effect and CCC between CDS and LB groups. Measures included overall performance in ANT-R, each attention network effect (RT and accuracy), and CCC. For one-way ANOVAs with significant differences, *Post-hoc* comparisons used LSD *post-hoc* tests for multiple-comparison correction. Bayesian factor analysis with the default value of 0.5 as the prior probability was conducted for each ANOVA. A Bayesian factor (BF_10_) larger than 3 indicates strong evidence for the alternative hypothesis, while smaller than 1/3 indicates strong evidence for the null hypothesis. Bayesian factor analyses were performed by JASP 0.17.3.0 (RRID: SCR_015823).

Pearson correlation analyses examined the association between CDS/LB symptoms and cognitive control abilities across the entire sample, controlling age and sex as covariates. Fisher’s z transform compared correlation coefficients. CDS symptoms were estimated based on the CCI-2 data, while the LB level was computed from the ASBI data. LB level was controlled when analyzing the association between the CDS symptoms and cognitive control abilities, and vice versa. To explore the relationships between the CCC and the attentional networks, Pearson correlation analyses were conducted across the sample finishing two tasks, controlling for age, sex, the CDS symptoms, and the LB level as covariates. Bayesian factor analysis was performed for each correlation.

### 2.5 Support vector machine modeling

Support vector machine modeling (SVM) was employed to assess whether CDS and LB could be distinguished based on their attention and cognitive control performance. We chose the Support Vector Machine (SVM) as our modeling method primarily due to its suitability for binary classification tasks, strong ability to avoid overfitting, good generalization capability, and appropriateness for small to medium-sized datasets, which are closely aligned with addressing our research question. Six binary target variables were utilized: (1) CDS versus TD, (2) LB versus TD, and (3) CDS + LB versus TD to identify significant cognitive control deficits in CDS and LB individuals; (4) CDS versus LB to ascertain for distinct cognitive control deficits between the two groups; (5) CDS+LB versus CDS, and (6) CDS+LB versus LB to explore synergistic cognitive control deficits in individuals with the composite symptoms.

Feature selection was based on Pearson correlation analysis results, in which cognitive measures showed a significant correlation to symptoms were selected as SVM features. Three sets of predicting features were used for each SVM: (1) two attention features from the ANT-R (validity and disengaging effects in RT), which were significantly related to CDS and/or CCC; (2) the CCC, which was also significantly related to CDS; and (3) a combination of the two attention features and CCC (ANT-R + CCC). Each measure was standardized into Z-scores across all participants. The feature set 3’s classification performance was compared to the first two sets to test whether a combination of attention and capacity deficits of cognitive control could provide additional information to distinguish CDS and LB.

Five-fold cross-validation evaluated the performance of each binary classification. Data were randomly divided into 5 folds, with 4 as the training set and the remaining fold as the testing set. The SVM classifier was trained based on the training set, and the estimated model parameters were applied to the testing set to predict the group labels.

Classification performance was evaluated using accuracy, as well as receiver operating characteristic (ROC) analysis based on specificity and sensitivity. Specifically, the classification had four possible outcomes: true positive (TP), true negative (TN), false positive (FP), and false negative (FN). Accuracy, the primary measure, was calculated as (TP + TN)/(TP + FP + FN + TN); specificity as TN/(TN + FP) and sensitivity as TP/(TP + FN). The ROC curve plots the TP against the FP at different classification thresholds, with the area under the curve (AUC) summarizing the model’s overall predictive ability.

Feature weights were calculated to reflect the relative importance of each feature in the classification model. A positive weight indicates that as the eigenvalue of that feature increases, the probability of the sample being classified as a positive class (e.g., having CDS) also increases. Conversely, a negative weight indicates that the probability of the sample being classified as the negative class (e.g., not having CDS) increases as the eigenvalue of that feature increases. The magnitude of the weight represents the degree of importance, with larger absolute values indicating greater relevance in the classification process. A total of 1000 permutations of 5-fold cross validation were performed for each classification, with average outcomes measures computed.

Chance level performance was estimated with another 1000 permutations, randomly shuffled group labels. The mean empirical classification performance was compared to the distribution of chance level performance to evaluate its significance. For target variables with classification accuracies better than chance, one-tailed planned comparisons were conducted to test: (1) whether the classification accuracy using attention features significantly differed from the accuracy using the CCC feature; and (2) whether the classification accuracy using the combined feature set was significantly better than the accuracy using attention features or CCC feature alone. Feature weight indicated directional influence, with positive values suggesting a greater probability of a sample having a positive label and negative values suggesting a greater probability of a sample having a negative label. For each SVM, the first mentioned group was set to a positive label and the latter was a negative label (for example, in models distinguishing CDS versus TD, the CDS group was a positive label and the TD group was a negative label).

## 3 Results

### 3.1 Diagnostic informations

Diagnostic information for each group was summarized in **Table 1**. The Chi-square and ANOVA tests indicated that there were no significant differences in age and sex between the groups. A significant main effect of Group was observed for CDS scores in the CCI-2 (all *p*s <.001 with *BF*_10_ > 1000) through the one-way 4-level (Group: CDS, LB, CDS + LB, TD) ANOVA. *Post hoc* comparisons revealed that the CDS group and the CDS + LB group showed significantly higher CDS scores than the LB group and the TD group (all *p*s <.001 with *BF*_10_ > 1000), while the CDS group and the CDS+LB group did not significantly different in CDS scores (all *p*s >.05 with 1/3 < *BF*_10_ < 3). The LB group showed significantly higher CDS scores than the TD group (all *p*s <.001 with *BF*_10_ > 1000).

Similarly, a significant main effect of Group was found for the LB scores in the ASBI (all *p*s <.001 with *BF*_10_ > 1000) through the one-way ANOVA test. *Post-hoc* comparisons showed that the LB group and the CDS + LB group had significantly higher LB scores than the CDS group and the TD group (all *p*s <.001 with *BF*_10_ > 1000), while the LB group and the CDS+LB group did not significantly different in LB scores (all *p*s >.05 with 1/3 < *BF*_10_ < 3). The CDS group exhibited significantly higher LB scores than the TD group (all *p*s <.001 with *BF*_10_ > 1000).

### 3.2 The unique factor structure of CDS

To verify the unique factor structure of CDS, exploratory factor analysis was conducted on the 15 items of the CCI-2 (38) and the 16 items of the ASBI (39) from the initial screening using principal axis factoring with Varimax rotation, with the factor loadings shown in **Table 2** (only the factor loadings higher than 0.4 was listed). Based on the structure of the CCI-2 containing only one factor and the structure of the ASBI containing three factors, four primary factors were assigned, accounting for over 53.53% of the total variance. Factor 1 comprised all 15 items of the CCI-2. Factors 2, 3 and 4 included items of three dimensions of the ASBI respectively, demonstrating a unique factor structure of CDS different from LB.

**Table 2:** The factor structure of the cognitive disengagement syndrome and the learning burnout.

Item	Factor			
	1	2	3	4
CDS_I zone out or space out	.74			
CDS_My thinking seems slow or slowed down	.71			
CDS_My mind gets mixed up	.71			
CDS_I lose my train of thought	.68			
CDS_I stare off into space	.68			
CDS_My mind feels like it is in a fog	.66			
CDS_I get lost in my own thoughts	.66			
CDS_I am not very active	.65			
CDS_I have hard time putting my thoughts into words	.63			
CDS_I feel confused	.62			
CDS_I daydream	.62			
CDS_I get tired easily	.56		.45	
CDS_I feel sleepy or drowsy during the day	.55			
CDS_I am slow at doing things	.54			
CDS_I forget what I am going to say	.53			
LB_I often feel exhausted recently		.81		
LB_I felt extremely tired at the end of the day’s study		.80		
LB_I often feel exhausted at school		.74		
LB_I feel very empty and don’t know what to do		.44		
LB_I can’t learn whether I learn or not			.77	
LB_I study with a cynical attitude			.75	
LB_I don’t think studying makes sense to me			.71	
LB_I study so badly that I really want to give up			.54	
LB_I can’t get a sense of achievement in terms of study			.40	
LB_I can always cope with study problems easily				.83
LB_I can effectively solve problems in study				.80
LB_It is easy for me to master what I have learned				.77
LB_I can often achieve my goals				.69
LB_I can cope well with exams				.69
LB_I forget everything around me when I study				.52
LB_I can devote myself to study energetically				.51

Factor loadings <.4 were omitted for clarity.

### 3.3 Attention effects

**Figure 3:**
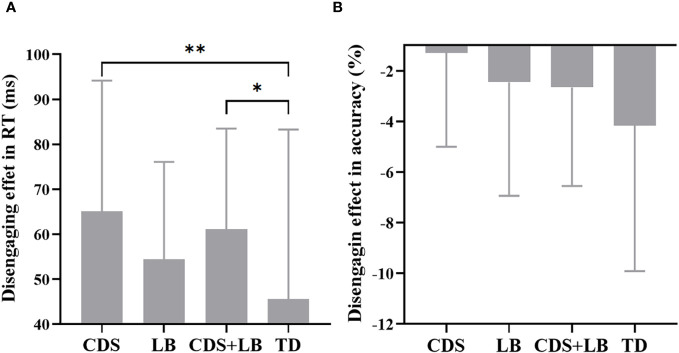
Disengaging effect between four groups. **(A)** The disengaging effect in RT for each group. **(B)** The disengaging effect in accuracy for each group. *CDS*, cognitive disengagement syndrome, *LB*, learning burnout, *TD*, typically developing peers, *CCC*, cognitive control capacity. *, *p* <.05; **, *p* <.01. Error bar indicates the standard deviation of the mean.

**Table 3** presents the attention network effects on RT and response accuracy in each group. The one-way ANOVA test revealed a significant main effect of Group was found only for the disengaging effect in RT (*F*_3,125_ = 2.89, *p* = .038, partial *η* ^2^ = .065, 1/3 < *BF*_10_ = 1.162 < 3; **Figure 3**), although not in accuracy (*F*_3,125_ = 2.18, *p* = .094, 1/3 < *BF*_10_ = 0.51 < 3). *Post-hoc* analyses revealed that the TD group showed a significantly smaller disengaging effect in RT compared to the CDS group (*p* = .007, 1/3 < *BF*_10_ = 2.275 < 3) and the CDS + LB group (*p* = .032, 1/3 < *BF*_10_ = 1.28 < 3), while there was no significant difference observed between the other pairs of groups (all *p*s >.05 with 1/3 < *BF*_10_ < 3). The main effect of Group was not significant for other measures: the overall RT and overall accuracy, the alerting effect in RT and accuracy, the orienting effect in RT and accuracy, the validity effect in RT and accuracy, the flanker conflict effect in RT and accuracy, the location conflict effect in RT and accuracy through one-way ANOVA tests.

**Table 3:** Mean ± SD of the attention network effects in each group.

Measure	Group				*F*	*p*	*BF_10_*
	CDS	LB	CDS+LB	TD			
Overall							
RT (ms)	628.1 ± 99.8	619.2 ± 74.9	617.23 ± 79.9	618.5 ± 70.7	< 1	.953	0.047
Accuracy (%)	94.1 ± 8.8	91.7 ± 11.3	92.5 ± 7.0	93.7 ± 8.7	< 1	.712	0.070
Alerting							
RT (ms)	53.2 ± 29.7	59.1 ± 27.7	53.3 ± 35.6	51.3 ± 35.6	< 1	.775	0.064
Accuracy (%)	-1.1 ± 3.7	-0.2 ± 4.4	-0.4 ± 6.2	-2.1 ± 6.0	1.05	.375	0.141
Orienting							
RT (ms)	43.8 ± 25.1	47.4 ± 20.4	57.4 ± 22.5	51.7 ± 29.1	1.66	.179	0.269
Accuracy (%)	0.1 ± 2.5	-0.7 ± 3.6	-1.5 ± 5.3	0.2 ± 3.5	1.39	.25	0.201
Validity							
RT (ms)	108.9 ± 40.9	101.9 ± 33.9	118.6 ± 30.6	97.4 ± 36.8	2.13	.1	0.475
Accuracy (%)	-1.1 ± 3.4	-3.2 ± 5.3	-4.1 ± 3.9	-3.9 ± 5.2	2.67	.051	0.841
Disengaging							
RT (ms)	65.1 ± 29.1	54.5 ± 21.6	61.2 ± 22.3	45.6 ± 37.7	2.89	.038^*^	1.162
Accuracy (%)	-1.3 ± 3.7	-2.5 ± 4.5	-2.7 ± 3.9	-4.2 ± 5.7	2.18	.094	0.51
Flanker conflict							
RT (ms)	104.1 ± 32.2	108.8 ± 40.7	109.6 ± 23.3	117.5 ± 47.2	< 1	.537	0.097
Accuracy (%)	-5.8 ± 7.8	-10.5 ± 17.7	-6.9 ± 5.5	-9.1 ± 15.8	< 1	.5	0.103
Location conflict							
RT (ms)	-6.3 ± 25.3	-6.8 ± 28.8	-4.2 ± 18.6	-6.6 ± 22.9	< 1	.973	0.045
Accuracy (%)	-0.6 ± 3.8	0.6 ± 3.9	-1.4 ± 2.7	0.3 ± 2.8	2.13	.1	0.469

RT, reaction time; CDS, cognitive disengagement syndrome; LB, learning burnout; TD, typically developing peers. *, p <.05.

### 3.4 Cognitive control capacity

**Figure 4:**
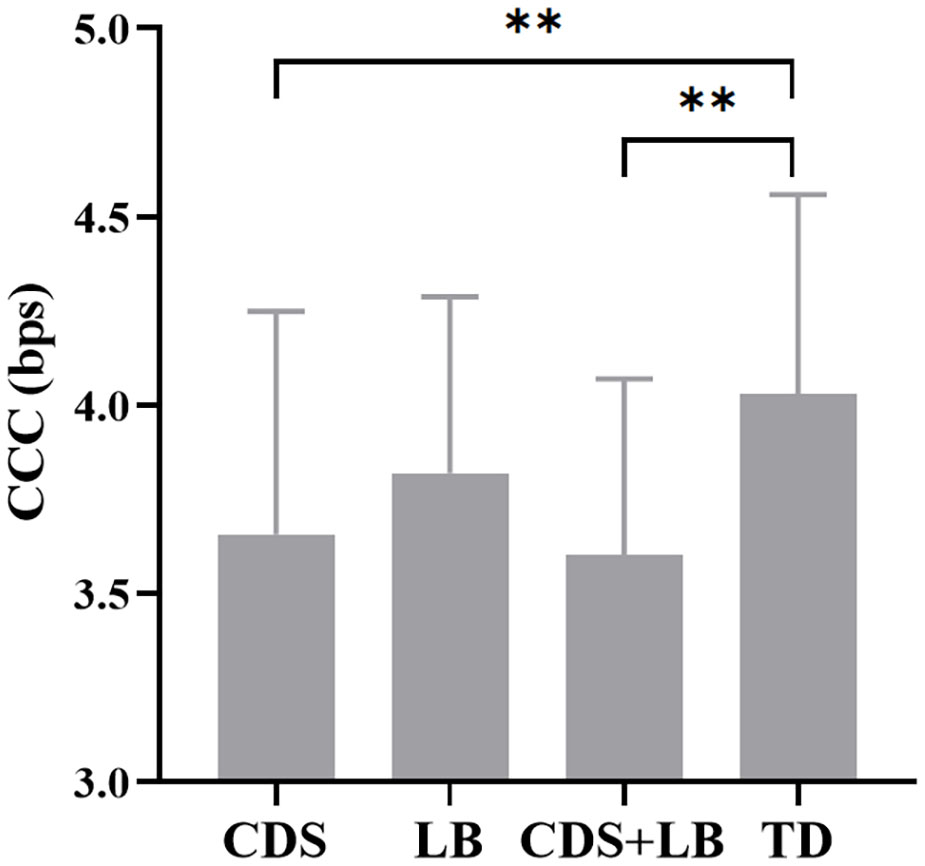
The CCC in each group. *CDS* = cognitive disengagement syndrome; *LB*, learning burnout; *TD*, typically developing; *CCC*, cognitive control capacity. **, *p* <.01. Error bar indicates the standard deviation of the mean.

The estimated CCC for each group is shown in **Figure 4**. The mean ± standard deviations of CCC (in bps) in the four groups were: CDS = 3.66 ± 0.59, LB = 3.82 ± 0.47, CDS + LB = 3.60 ± 0.47, TD = 4.03 ± 0.53. The one-way ANOVA test revealed a significant main effect of Group was significant (*F*_3,113_ = 4.46, *p* = .005, partial *η* ^2^ = .106, *BF*_10_ = 7.881 > 3), with the TD group showing significantly higher CCC compared to the CDS group (*p* = .004, *BF*_10_ = 5.605 > 3) and the CDS+LB group (*p* = .002, *BF*_10_ = 22.257 > 3), while there was no significant difference observed between the other pairs of groups (all *p*s >.05 with 1/3 < *BF*_10_ < 3).

### 3.5 Correlation analysis

Results of the Pearson correlation analysis between the CDS/LB symptoms and attention effects in RT/CCC are reported in **Table 4**. We found a significant positive correlation between the disengaging effect in RT and the CDS scores (*r* = .202, *p* = .023) when controlling LB scores as a covariate. The CCC was significantly negatively related to the CDS scores (*r* = -.233, *p* = .013) when adding LB scores as a covariate, while the CCC was not significantly related to the LB scores when considering CDS scores as a covariate. Other correlation coefficients were not significant.

**Table 4:** Correlations coefficients between CDS symptoms, LB symptoms, attention network effects in RT and CCC.

	CDS	LB	CCC	Fisher’ s *Z*	*p*
Overall RT	-.029	.027	-.014	-0.446	.655
Alerting	-.067	.090	-.207	-1.253	.21
Orienting	-.043	.064	-.032	-0.854	.393
Validity	.135	.036	-.263*	0.795	.426
Disengaging	.202*	-.012	-.279*	1.728	.084
Flanker conflict	-.065	-.067	.048	0.016	.987
Location conflict	-.034	-.058	.104	0.192	.848
CCC	-.233*	-.100	–	-1.034	.301

CDS, cognitive disengagement syndrome; LB, learning burnout; CCC, cognitive control capacity; RT, reaction time. *: p <.05.

Fisher’s-z transforms were conducted to further examine the between-group (CDS vs. LB) difference of each correlation coefficient. We did not find a significant between-group difference in the correlation between attention efficiency/CCC and symptom scores.

Furthermore, the CCC was significantly negatively related to the validity effect in RT (*r* = -.263, *p* = .017) and the disengaging effect in RT (*r* = -.279, *p* = .011) when controlling CDS scores and LB scores as covariates, while no other significant correlation was found.

### 3.6 Support vector machine modeling

**Figure 5:**
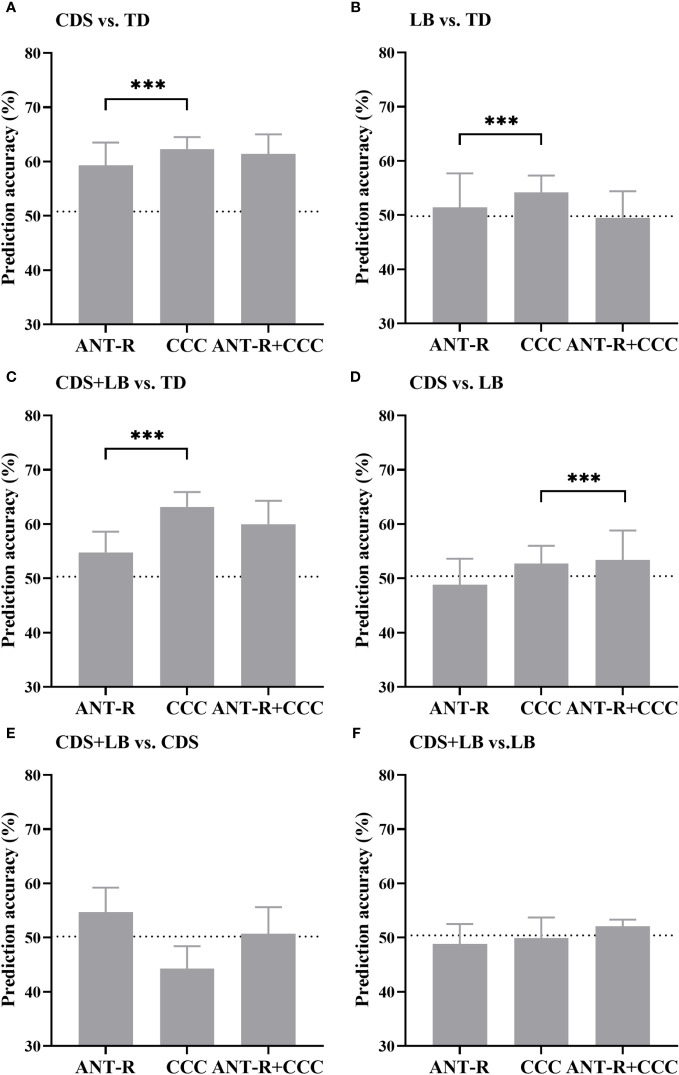
Prediction accuracy of all models. **(A)** The prediction accuracy of CDS vs. TD. **(B)** The prediction accuracy of LB vs. TD. **(C)** The prediction accuracy of CDS+LB vs. TD. **(D)** The prediction accuracy of CDS vs. LB. **(E)** The prediction accuracy of CDS+LB vs. CDS. **(F)** The prediction accuracy of CDS+LB vs. LB. *CDS*, cognitive disengagement syndrome; *LB*, learning burnout; *TD*, typical developed peers. *ANT-R (x-axis)* indicates the model with combined of validity and disengaging effects in RT as the features, *CCC (x-axis)* indicates the model with the cognitive control capacity as the feature, *ANT-R+CCC (x-axis)* indicates the model combined of both validity and disengaging effect in RT and cognitive control capacity. Dotted lines indicate chance levels. Error bar indicates the standard deviation of the mean.

**Figure 6:**
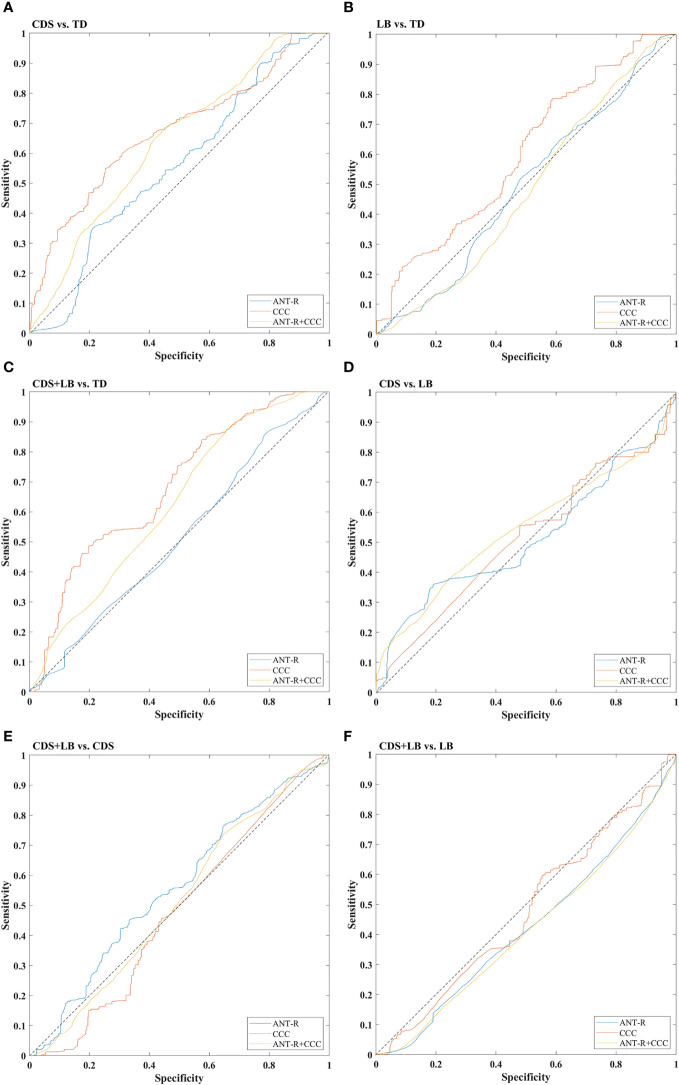
ROC curves of all models. **(A)** The ROC curves of CDS vs. TD. **(B)** The ROC curves of LB vs. TD. **(C)** The ROC curves of CDS+LB vs. TD. **(D)** The ROC curves of CDS vs. LB. **(E)** The ROC curves of CDS+LB vs. CDS. **(F)** The ROC curves of CDS+LB vs. LB. *CDS*, cognitive disengagement syndrome; *LB*, learning burnout; *TD*, typical developed peers. *ANT-R (curve)* indicates the model with combined of validity and disengaging effects in RT as the features, *CCC (curve)* indicates the model with the cognitive control capacity as the feature, *ANT-R+CCC (curve)* indicates the model combined of both validity and disengaging effect in RT and cognitive control capacity. Dotted lines indicate chance level.

**Table 5** presents the specificity, sensitivity, and AUC values for each model, demonstrating their overall unbiased performance. The prediction accuracies are shown in **Figure 5** and **Table 6**, and the ROC curves visualizing the classification power of each model are depicted in **Figure 6**. The feature weights of each model are shown in **Table 7**.

**Table 5:** Mean ± SD of the specificity, sensitivity and the area under the ROC curve of each model.

Comparison	Feature set	Specificity	Sensitivity	AUC
CDS vs. TD	ANT-R	23.4 ± 5.7%	89.2 ± 6.0%	56.1 ± 4.6%
	CCC	55.7 ± 3.8%	67.9 ± 3.1%	67.0 ± 1.4%
	ANT-R+CCC	50.2 ± 5.3%	70.8 ± 4.4%	63.8 ± 3.6%
LB vs. TD	ANT-R	47.3 ± 8.4%	55.5 ± 8.1%	49.0 ± 6.3%
	CCC	52.7 ± 3.3%	55.7 ± 5.7%	60.3 ± 2.2%
	ANT-R+CCC	43.0 ± 7.4%	56.0 ± 7.3%	47.8 ± 5.4%
CDS+LB vs. TD	ANT-R	18.7 ± 7.0%	87.7 ± 4.2%	52.2 ± 4.0%
	CCC	49.3 ± 3.6%	75.6 ± 4.6%	68.2 ± 1.3%
	ANT-R+CCC	46.2 ± 7.6%	72.5 ± 5.0%	62.7 ± 3.3%
CDS vs. LB	ANT-R	32.9 ± 9.0%	62.2 ± 8.0%	51.6 ± 4.4%
	CCC	29.0 ± 9.8%	72.4 ± 6.2%	51.4 ± 4.3%
	ANT-R+CCC	46.2 ± 8.8%	59.4 ± 8.0%	54.5 ± 5.4%
CDS+LB vs. CDS	ANT-R	56.2 ± 6.8%	53.0 ± 6.6%	55.6 ± 4.3%
	CCC	66.4 ± 12.3%	20.2 ± 9.5%	47.5 ± 5.4%
	ANT-R+CCC	49.8 ± 7.8%	51.7 ± 6.8%	51.2 ± 5.1%
CDS+LB vs. LB	ANT-R	6.3 ± 5.0%	87.5 ± 8.5%	41.9 ± 5.5%
	CCC	34.8 ± 10.6%	63.7 ± 6.8%	48.2 ± 5.0%
	ANT-R+CCC	0.2 ± 1.1%	99.2 ± 2.6%	41.6 ± 5.3%

CDS, cognitive disengagement syndrome; LB, learning burnout; TD, typical developed peers; ANT-R, features as validity and disengaging effects in RT of the attentional network test-revised; CCC, features as cognitive control capacity; ANT-R+CCC, features as both validity and disengaging effects in RT of the attentional network test-revised and cognitive control capacity; AUC, area under the ROC curve.

**Table 6:** Mean ± SD of the prediction accuracy of each model.

Comparison	Chance level	ANT-R	CCC	ANT-R+CCC
CDS vs. TD	50.8 ± 7.9%	59.3 ± 4.2%	62.3 ± 2.2%	61.4 ± 3.6%
LB vs. TD	49.8 ± 8.0%	51.4 ± 6.3%	54.2 ± 3.1%	49.5 ± 4.9%
CDS+LB vs. TD	50.3 ± 7.9%	54.8 ± 3.8%	63.1 ± 2.8%	60.0 ± 4.3%
CDS vs. LB	50.4 ± 7.7%	48.8 ± 4.8%	52.7 ± 3.3%	53.4 ± 5.4%
CDS+LB vs. CDS	50.2 ± 7.0%	54.7 ± 4.5%	44.3 ± 4.1%	50.7 ± 4.9%
CDS+LB vs. LB	50.4 ± 7.8%	48.8 ± 3.7%	49.9 ± 3.8%	52.1 ± 1.2%

CDS, cognitive disengagement syndrome; LB, learning burnout; TD, typical developed; ANT-R, features as validity and disengaging effects of the attentional network test-revised; CCC, features as cognitive control capacity; ANT-R+CCC, features as both validity and disengaging effects in RT of the attentional network test-revised and cognitive control capacity.

**Table 7:** The feature weights of each model.

Feature (P vs. N)	Feature set	Validity	Disengagement	CCC
CDS vs. TD	ANT-R	-0.369 ± 0.059	0.858 ± 0.053	
	CCC			-0.0221 ± 0.000
	ANT-R+CCC	-1.304 ± 0.216	2.144 ± 0.188	-2.213 ± 0.114
LB vs. TD	ANT-R	0.396 ± 0.081	0.767 ± 0.074	
	CCC			-0.068 ± 0.003
	ANT-R+CCC	0.014 ± 0.015	0.038 ± 0.015	-0.104 ± 0.007
CDS+LB vs. TD	ANT-R	0.097 ± 0.008	0.099 ± 0.006	
	CCC			-31.906 ± 0.0823
	ANT-R+CCC	23.105 ± 1.286	20.917 ± 1.076	-54.372 ± 1.560
CDS vs. LB	ANT-R	-0.286 ± 0.046	0.336 ± 0.041	
	CCC			-0.049 ± 0.011
	ANT-R+CCC	-0.722 ± 0.059	0.722 ± 0.053	-0.494 ± 0.047
CDS+LB vs. CDS	ANT-R	0.367 ± 0.033	-0.363 ± 0.029	
	CCC			0.026 ± 0.008
	ANT-R+CCC	0.685 ± 0.045	-0.598 ± 0.045	0.211 ± 0.040
CDS+LB vs. LB	ANT-R	0.054 ± 0.011	0.011 ± 0.009	
	CCC			-0.043 ± 0.008
	ANT-R+CCC	0.034 ± 0.006	0.003 ± 0.005	-0.046 ± 0.005

P, positive class; N, negative class; CDS, cognitive disengagement syndrome; LB, learning burnout; TD, typical developed peers; ANT-R, features as validity and disengaging effects of the attentional network test-revised; CCC, features as cognitive control capacity; ANT-R+CCC, features as both validity and disengaging effects in RT of the attentional network test-revised and cognitive control capacity.

For the CDS versus TD classification (**Figure 5A**), all feature sets yielded prediction accuracies significantly better than chance level (all *p*s <.001). Planned comparisons showed that the prediction accuracy using the CCC feature was significantly higher than using the attention features (*p* <.001), while using the combined features did not significantly enhance the accuracy compared to using the CCC feature alone (*p* >.999). These results indicate that the CCC provided unique information to distinguish CDS from TD, with CDS exhibiting a defect in CCC.

For the LB versus TD classification (**Figure 5B**), the attention features and the CCC feature yielded prediction accuracies significantly better than chance level (all *p*s<.001). Planned comparisons showed that the prediction accuracy using the CCC feature was significantly higher than using the attention features (*p* <.001). These results suggest that CCC contributed to distinguishing LB from TD, with deficit in CCC possibly indicating inclusion in the LB category.

For CDS + LB versus TD classification (**Figure 5C**), all feature sets yield prediction accuracies significantly better than chance level (all *p*s <.001). Planned comparisons showed that the prediction accuracy using the CCC feature was significantly higher than using the attention features (*p* <.001), while using the combined features did not significantly enhance the accuracy compared to using the CCC feature alone (*p* >.999). These results indicate that compared to TD, CDS + LB had a synergistic impact on CCC.

For the CDS versus LB classification (**Figure 5D**), the CCC feature and the combined features yielded prediction accuracies significantly better than chance level (all *p*s <.001). Planned comparisons showed that using the combined features significantly enhanced the accuracy compared to using the CCC feature alone (*p* <.001). These results suggest that the CCC feature provided unique information to distinguish CDS and LB, with CDS exhibiting a lower CCC, while the attention features could provide information only when combined with CCC.

For the CDS + LB versus CDS classification (**Figure 5E**), the attention features and the combined features yield prediction accuracies significantly better than chance level (attention: *p* <.001, combined: *p* = .04). Planned comparisons showed using the combined features did not significantly enhance the accuracy compared to using the attention features alone (*p* >.999). These results indicate that compared to the CDS, CDS + LB had a synergistic impact on the attention networks.

For the CDS + LB versus LB classification (**Figure 5F**), only the combined features could yield prediction accuracies significantly better than chance level (*p* <.001). In addition, the recall scores indicated that the model was biased, rendering them inadequate for accounting for the results. These results indicate that compared to the CDS, CDS + LB had a synergistic impact on the attention networks.

## 4 Discussion

In this study, we delved into cognitive control deficits among individuals with CDS and LB, generally aligning our findings with our initial hypotheses. The exploratory factor analysis demonstrated the cross-cultural stability of the CDS as a distinct construct, further clarifying this structural distinction from LB. Our results suggest that adolescents exhibiting CDS symptoms are prone to cognitive control impairments, particularly in attention disengagement and cognitive control capacity. Interestingly, LB individuals did not manifest comparable impairments when compared to TD peers. Employing SVM analyses, we unveiled a synergistic impact of CDS and LB on CCC, highlighting the intricate interplay of cognitive control mechanisms with both conditions. Furthermore, SVM analyses emphasized the pivotal role of CCC in distinguishing between CDS and LB, elucidating their distinct cognitive profiles.

### 4.1 Impaired attention orienting function

The heightened disengaging effect observed in adolescents with CDS, compared to TD counterparts, underscores their ability to redirect attention from distracting stimuli. However, the Bayesian factor indicated that the evidence supporting the disengagement effect in distinguishing between CDS and TD was relatively weak, suggesting that this finding needs to be further validated. This finding aligns with previous research revealing impaired attention orienting function in young adults (17). Conversely, investigations in children aged 7 to 10 years found that while CDS was associated with delayed response time, significant impacts on attention networks were not observed (30, 31), highlighting the age-dependent nature of cognitive functions and CDS-related problem in orienting, which emerge in adolescence and persisting in early adulthood (41, 44).

The continuous model of activity suggests that the inattention may exhibit an inverted U-shaped relationship with the activity level (45). Described as slow and underactive, CDS has been shown to be associated with poor performance in attention tasks, particularly in early selective attention, sustained attention and attention shifting (1, 32). Studies have reported that CDS adolescents exhibit reduced response in the left superior parietal lobe (46), a region related to the orienting network (47). The diminished activity in the superior parietal lobe may explain the observed orienting and disengaging effects in the previous study (17) and our current findings. CDS symptoms have been linked to overactivity in inhibition (35), potentially hindering attention switching from current stimuli to target stimuli, thereby resulting in a poor disengaging effect.

### 4.2 Deficits in cognitive control

Our study revealed that adolescents with CDS symptoms exhibited deficits in cognitive control, particularly concerning the capacity. Remarkably, both groups of adolescents with high CDS symptoms demonstrated a significantly lower level of CCC compared to the TD group. The Bayesian factor indicates that the results are supported by moderate to strong evidence. This diminished CCC was further associated with a heightened impact on the disengaging effect. The perceptual load theory (48) elucidates that excessive task demands can hinder the efficient processing of irrelevant information, thereby affecting resources allocation and subsequent task performance. The compromised disengagement performance in high CDS adolescents may stem from their constrained CCC, limiting their resources for pausing the ongoing activities (e.g., focusing on a list box) and detecting novel stimulus (e.g., appearing in another box), evidenced by the negative association between CCC and disengagement effect observed in this study. Although cognitive control deficits were associated with CDS symptoms in our study, these deficits alone could not fully account for CDS, as they only partially explained the symptomatology, showed modest correlations and predictive utility for CDS, and are transdiagnostic impairments observed across multiple neurodevelopmental and emotional disorders.

The results did not entirely align with the general hypothesis that the adolescents with high CDS symptoms would exhibit deficits across attention functions. The present findings indicated that high CDS symptoms were specifically associated with deficits in disengagement of attention, rather than a broad impairment across all attentional components. However, these disengagement deficits were related to reductions in the core cognitive control capacity, as evidenced by lower CCC scores. It is possible that impairments in this foundational capacity contribute to the overactivity of the inhibitory ability observed in CDS, leading to a reduction in information input and difficulties with attention switching, manifested as deficits in disengagement. Heightened task-independent thoughts in CDS individuals may divert cognitive resources away from the required task, contributing to reduced attention towards external stimuli (49). This immersion in internal thoughts may explain their seemingly reduced interest in the external world (1). Additionally, another fMRI study found that CDS symptoms were associated with an increase in the regional volume of specific frontal regions crucial for general executive function, suggesting a potential association of CDS with high-order cognitive processes (50), such as cognitive control capacity.

### 4.3 CCC as the main distinguishing factor

The SVM models showed that CCC was the most crucial feature in distinguishing CDS from LB, and the attention features could be helpful only when combined with the CCC feature. These results suggested that deficits in cognitive control, specifically in the CCC, may differentiate between individuals with high CDS and LB symptoms. Our findings indicated a significant reduction in CCC among individuals with high CDS symptoms but not among those with high LB symptoms. CCC reflects the information processing efficiency of cognitive control, a fundamental and indispensable cognitive process. The unique impact of CDS on CCC may suggest that CDS, as a trait-like disorder (3), exerts a more profound and pervasive negative effect on adolescents than LB, which may represent a more a state-like issue. The effects of CDS appear to extend to core cognitive abilities, such as attention and cognitive control. In contrast, LB represents an attitude or response towards specific situational stressors like academic pressure, without necessarily impacting fundamental cognitive capacities. Consequently, while psychological and educational interventions can be beneficial for ameliorating LB (13), CDS may require more specialized clinical treatment approaches, potentially involving pharmacotherapy, as it represents a clinical condition rather than solely a behavioral problem (11).

This result may be attributed to the non-clinical nature of our LB samples. Previous studies have reported inconsistent impacts of burnout on cognitive control-related functions, likely due to variations in the relationship between burnout symptoms and cognitive performance across clinical and non-clinical contexts (27, 28), as well as across different stages of clinical contexts (51). A study directly comparing clinical and non-clinical burnout revealed that while both clinical burnout and non-clinical burnout individuals subjectively reported more cognitive problems than the healthy participants, only clinical burnout patients objectively displayed impaired cognitive performance as assessed by a series of cognitive tests (52). Consistent with the result, studies found that non-clinical burnout was not sufficient to affect cognitive performance (26, 53), while a meta-analysis showed that clinical burnout was associated with small to moderate impairments of executive function in neuropsychological tests (27).

Moreover, we suspect that burnout-related cognitive impairments may be context-specific. Learning burnout symptoms are specific to activities in school, characterized by a lack of interest in learning and poorer performance only on learning-related tasks, while individuals may remain active in other unrelated activities (54). Since the cognitive tasks utilized in the current study were not directly related to learning, they may not have been sensitive to detecting symptoms of LB. In contrast, the cognitive deficits observed in CDS may be more general. Adolescents with high CDS experience distraction and disinterest in everything, irrespective of its relevance to learning (30). Therefore, it is plausible that cognitive control, in a domain- and task-general context, represents a key distinction between adolescents with CDS and those with LB in the non-clinical context. It is important to note that our sample consisted solely of non-clinical adolescents, and therefore, potential cognitive differences between clinical and non-clinical CDS populations were not examined in the current study.

### 4.4 Limitations

This study has several limitations that should be acknowledged. Firstly, the sample may not be sufficiently large and representative, potentially impacting the robustness of our findings. Larger samples would enhance the generalizability and reliability of the research outcomes. Moreover, our participants were exclusively recruited from school settings, which may limit the generalizability of our findings to clinical populations diagnosed with CDS in hospital settings, as clinical presentations of CDS may differ from those observed in community or school environments (1). Future research could explore whether similar patterns are observed in clinical populations. Secondly, another limitation is that we did not assess for comorbid attention deficits/hyperactivity disorder (ADHD), autism spectrum disorder (ASD), and depression, which frequently co-occur with CDS (1). These conditions share overlapping symptoms with CDS, such as inattention, social difficulties, and emotional dysregulation. By not accounting for the presence and severity of these comorbidities, we cannot rule out that the observed cognitive and functional impairments in our CDS sample may be partly attributable to them. Further research directly comparing the cognitive control profiles of individuals with CDS to those with ADHD would help clarify whether the deficits observed represent a shared or distinct impairment between the two conditions. Future studies should include comprehensive assessments to delineate the unique cognitive profiles of CDS while controlling for coexisting neurodevelopmental and psychiatric disorders, enhancing the specificity and clinical utility of the findings. Additionally, the potential influence of age on the relationship between CDS and the attentional networks warrants further investigation. Although previous studies have explored this relationship across different age groups, inconsistencies exist due to variations in experimental tasks, necessitating more focused investigations. Lastly, given the behavioral similarities observed between individuals with CDS and those with LB, further research could explore whether CDS may contribute to the development of LB, and whether the presence of LB signifies a heightened degree of CDS symptoms. Exploring these associations could provide valuable insights into the underlying mechanisms of both conditions and inform targeted interventions.

## 5 Conclusion

In conclusion, this study sheds light on cognitive control deficits among adolescents exhibiting symptoms of CDS, particularly evident in impaired disengaging of attention and a decrease in cognitive control capacity. Our findings also suggest that cognitive control capacity could serve as a valuable parameter for differentiating between CDS and LB. This research contributes to the understanding of how CDS impacts attention and cognitive control in adolescents, providing a foundation for future investigations aimed at optimizing diagnostic criteria for CDS. Moreover, our findings carry practical implications for clinical therapy, school intervention, and family support tailored to individuals with CDS and LB. Despite exhibiting similar outward manifestations, these conditions may require distinct intervention strategies (11, 13). Establishing reliable criteria to distinguish between CDS and LB is crucial for effective intervention strategies targeting these symptoms.

## Data availability statement

The raw data supporting the conclusions of this article will be made available by the authors, without undue reservation.

## Ethics statement

The studies involving humans were approved by the Human Research Ethics Committee for Non-Clinical Faculties, the School of Psychology, South China Normal University. The studies were conducted in accordance with the local legislation and institutional requirements. Written informed consent for participation in this study was provided by the participants’ legal guardians/next of kin.

## Author contributions

YHW: Conceptualization, Formal Analysis, Methodology, Software, Writing – original draft. TW: Methodology, Software, Writing – review & editing. YFW: Data curation, Investigation, Writing – original draft. LC: Visualization, Writing – original draft. XL: Writing – original draft, Writing – review & editing. KC: Writing – original draft. CC: Conceptualization, Funding acquisition, Writing – review & editing.
